# Comparison of Analytical Sensitivity (Limit of Detection) of Xpert MTB/RIF and Xpert MTB/RIF Ultra for Non-Sputum Specimens

**DOI:** 10.3390/pathogens12020157

**Published:** 2023-01-18

**Authors:** Marisa C. Nielsen, Paula Clarner, Ruchi Paroha, Sunhee Lee, Phyu M. Thwe, Ping Ren

**Affiliations:** 1 Department of Pathology, University of Texas Medical Branch, Galveston, TX 77555, USA; 2 Department of Immunology and Microbiology, University of Texas Medical Branch, Galveston, TX 77555, USA; 3 Department of Pathology, Infectious Disease Testing, Montefiore Medical Center, Bronx, NY 10467, USA

**Keywords:** analytical sensitivity, limit of detection (LoD), *Mycobacterium tuberculosis*, extrapulmonary, non-sputum specimens

## Abstract

Tuberculosis (TB) is a significant public health threat and has remained a leading cause of death in many parts of the world. Rapid and accurate testing and timely diagnosis can improve treatment efficacy and reduce new exposures. The Cepheid Xpert® MTB/RIF tests have two marketed products (US-IVD and Ultra) that are widely accepted for diagnosis of TB but have not yet been approved for non-sputum specimens. Despite numerous studies in the literature, no data for the analytical sensitivity of these two products on the non-sputum samples are available to date. This is the first study that systematically determined the analytical sensitivities of both US-IVD and Ultra tests on cerebrospinal fluid (CSF), tissue, and bronchoalveolar lavage (BAL). The limits of detection (LoDs) on the US-IVD test for both *Mycobacterium tuberculosis* and rifampin resistance in CFU/mL, respectively, were as follows: CSF (3.3 and 4.6), tissue (15 and 23), and bronchoalveolar lavage (BAL) (45 and 60), and on the Ultra test: CSF (0.16 and 2.7), tissue (0.11 and 12), and BAL (0.65, and 7.5). Overall, the analytical sensitivities of the Ultra test were substantially better than US-IVD for all sample types tested. This study provided a foundation for using either the US-IVD or Ultra test for the early detection of both pulmonary and extrapulmonary (EP) TB. Furthermore, using Ultra could result in higher TB case detection rates in subjects with paucibacillary TB and EP TB, positively impacting WHO goals to eradicate TB.

## 1. Background

*Mycobacterium tuberculosis* (MTB) remains a significant cause of morbidity and mortality worldwide. Even though MTB is ranked as one of the leading causes of death by a single infectious agent, over the past decade, there had been a slowly declining trajectory in cases globally [1]. However, that was substantially reversed by the COVID-19 pandemic due to a reduction in healthcare access, close household contact during lockdowns, and testing, even in the setting of increased mask use and other public health initiatives to mitigate the spread of SARS-CoV-2 [1].

Early and rapid detection of MTB and rifampin (RIF) resistance is crucial to help prevent transmission and overcome significant treatment challenges associated with drug-resistant MTB. The gold standard for MTB diagnosis is currently culture-based. Since MTB grows slowly and identification capabilities may be limited in many laboratories, the Cepheid Xpert® MTB/RIF test performed on the GeneXpert® system (Cepheid, Sunnyvale, CA, USA) is widely accepted for the initial diagnosis of suspected pulmonary tuberculosis (TB) [2,3]. Even though major public health strategies have been designed to mitigate the spread of MTB, an estimated 1/3 of all cases remain undiagnosed [4]. MTB in extrapulmonary (EP) sources accounted for most undiagnosed or delayed diagnosed cases. Although EP TB is not as prevalent as pulmonary TB in the United States, some forms of EP TB, such as meningeal or spinal TB, can be devastating and life-threatening [5,6,7].

Xpert® MTB/RIF (US-IVD) is an in vitro diagnostic (IVD) test in the US that can simultaneously detect MTB DNA and RIF-resistance-associated mutations of the *rpoB* gene only from sputum samples. It is a qualitative, nested real-time polymerase chain reaction (PCR) test in which the primers are designed to amplify the 81 base pair core region of the *rpoB* gene, also called the RIF resistance-determining region (RRDR). In addition, five probes are designed to differentiate the conserved wild-type sequence and mutations in the RRDR [8,9]. They are Probe A for codons 507–511, Probe B for codons 511–518, Probe C for codons 518–523, Probe D for codons 523–529, and Probe E for codons 529–533 [10].

Xpert® MTB/RIF Ultra (Ultra) is approved by the European Medicines Agency (EMA) for IVD use on unprocessed sputum samples or concentrated sediments prepared from induced or expectorated sputum. However, it has neither been approved by US Food and Drug Administration (FDA), nor is it available in the US. Unlike the US-IVD test, Ultra is a semi-quantitative, nested real-time PCR test with melt peak detection. The Ultra test primers amplify the same core region of the *rpoB* gene as the US-IVD test, but with four probes. Mutations in the RRDR are captured by a shift in the melting temperature (Tm) of at least one of the four probes away from the Tm expected to occur in the presence of wild-type [11]. In addition, the primers in the Ultra test amplify portions of the multi-copy insertion elements *IS1081* and *IS6110*, which enhance the detection of MTB [12]. Ultra has been documented to be more sensitive than US-IVD, but may be less specific based on the disease prevalence and underlying comorbidities [13,14].

Currently, US-IVD is FDA approved for use on sputum only and has a documented LoD of 131 CFU/mL [15]. Ultra has demonstrated high sensitivity/specificity for MTB and RIF-resistance detection in patients with pulmonary TB [3]. Chakravorty et al. reported the improved detection of MTB and RIF resistance by Ultra with an LoD of 15.6 CFU/mL of sputum [8]. However, EP TB can be notoriously challenging to diagnose, partly due to low concentrations of the organism. This study aimed to compare the analytical sensitivities of both US-IVD and Ultra on non-sputum samples.

## 2. Methods

### 2.1. Preparation of MTB Stock

An RIF-resistant MTB clinical isolate 10330068 was subcultured on a Lowenstein–Jensen Medium (LJ) agar slant (Thermo Fisher Scientific, Waltham, MA, USA) for 12–15 days at 36 °C. The organism was suspended in sterile saline and adjusted to 1 McFarland (0.39 OD_600_) to obtain an estimated stock concentration of 1.97 × 10^6^ CFU/mL [16].

### 2.2. Sample Preparation

MTB-negative bronchoalveolar lavage (BAL), cerebrospinal fluid (CSF), and lymph node tissue specimens from TB culture-negative patients were pooled based on sources. After pooling, pooled samples from each source were tested by US-IVD and Ultra to ensure MTB was not detectable. These pooled MTB-negative specimens were used as matrices for each source analyzed. Lymph node tissue was mixed with sterile phosphate-buffered saline (PBS) (1:1) and ground using a sterile tissue grinder. The MTB–saline stock was spiked into one portion of the appropriate pooled specimens. Then, 1:2 serial dilutions were performed using the other portions to obtain representative concentrations ranging from 3940 colony forming units per mL (CFU/mL) to 0.004 CFU/mL and 18–22 replicates of each concentration (636 total tests) were frozen at −80 °C until tested. About ten each were used for US-IVD and Ultra tests, respectively. To further determine the CFU/mL for each specimen type, 100 μL of the suspensions with expected concentrations between 25–150 CFU/mL were plated on Middlebrook 7H11 agar plates (Thermo Scientific^TM^ Remel^TM^, San Diego, CA, USA) in triplicate. After 3 weeks of incubation at 36 °C, colonies were counted. MTB concentrations were calculated based on colony counts for each specimen type.

### 2.3. Xpert MTB/RIF US-IVD and Xpert MTB/RIF Ultra Tests

Aliquots were treated with a 1:1 Sample Reagent provided in the testing kits to sample volume for CSF [17] and 2:1 for BAL [18] and tissue [19], per the manufacturer’s guidelines for non-sputum specimens. After vigorous mixing and room temperature incubation for 15 min, 2 mL of the treated sample was loaded into either a US-IVD or Ultra cartridge and tested per the manufacturer’s instructions.

### 2.4. Statistical Analysis

Linear regressions (dose–response analysis) were performed for each specimen type tested by US-IVD and Ultra, respectively, using MedCalc® statistical software (MedCalc Software Ltd., Ostend, Belgium)

## 3. Results

### 3.1. Limit of Detection (LoD)

**Figure 1:**
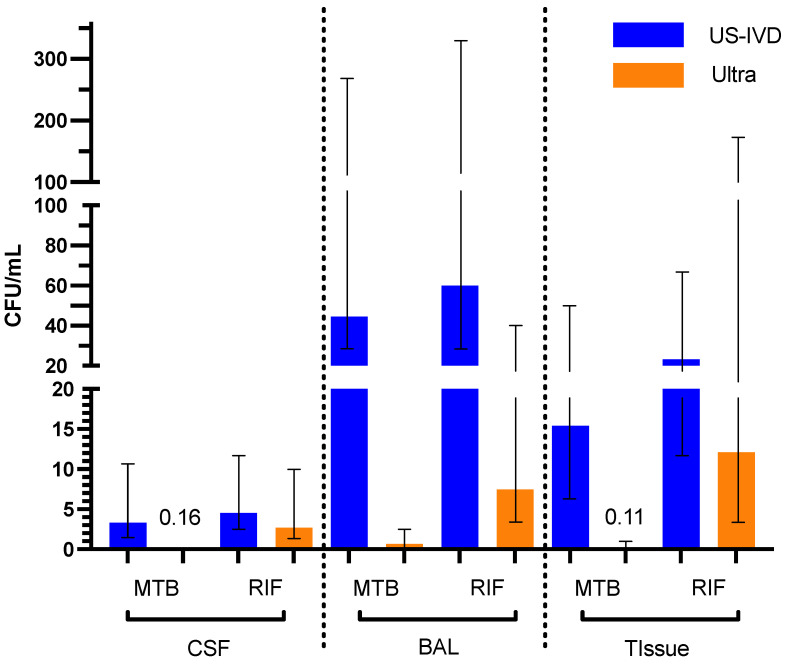
Limit of Detections between US-IVD and Ultra on different sample types for both MTB and RIF. The bars reflect the 95% confidence intervals.

The LoDs for MTB and RIF were determined for US-IVD and Ultra on all three sample types. The US-IVD test had the lowest LoDs for both MTB and RIF on CSF (3.3 CFU/mL and 4.6 CFU/mL, respectively) and the highest LoDs for both MTB and RIF on BAL (45 CFU/mL and 60 CFU/mL, respectively) among these three sample types. In comparison, the Ultra test had the lowest LoD for MTB (0.11 CFU/mL), but the highest LoD for RIF (12 CFU/mL) on tissue samples among the three sample types (Table 1). The LoD of MTB on BAL was highest (0.65 CFU/mL) and the LoD of RIF on CSF (2.7 CFU/mL) was lowest for the Ultra test (Table 1). Overall, the analytical sensitivity of the Ultra test is significantly higher than that of US-IVD, regardless of the sample types (*p* < 0.05) (Table 1 and Figure 1).

### 3.2. Linearity for US-IVD and Ultra

**Figure 2:**
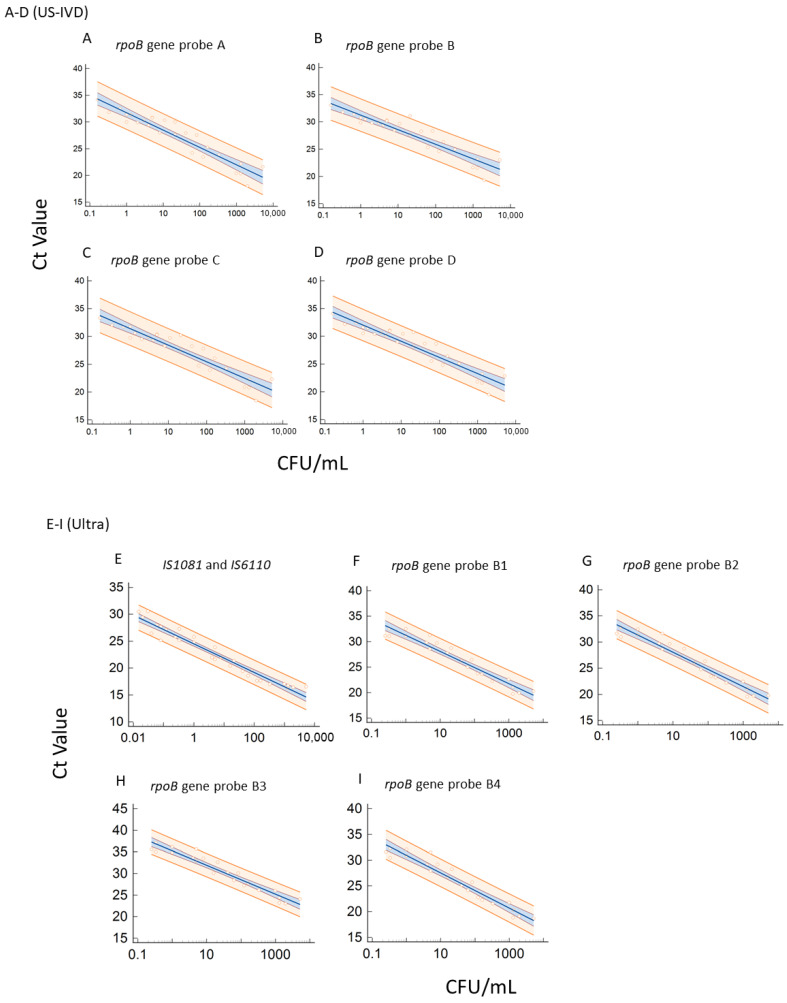
(**A**–**D**) Linear regression for the *rpoB* gene with detection probes A, B, C, and D (US-IVD), respectively. (**E**–**I**) Linear regression for the *IS* and the *rpoB* gene with detection probes B1, B2, B3, and B4 (Ultra), respectively. The orange area indicates the 95% confidence interval.

The *rpoB* gene was amplified and detected by five probes (A–E) in US-IVD, with the cycle threshold (Ct) set at 39 for A, B, and C, and 36 for D and E [9]. As expected, Ct values for each probe increased as the concentration decreased in all sample types (Figure 2A–D). Mutations associated with the Probe E (codons 529–533) region were identified as the most common *rpoB* gene mutations [10]. Based on the Ct values, the RIF-resistant MTB isolate used in this study had mutations in the probe E region of the *rpoB* gene. The software reported probe E target Ct values as 0 for all tests run on the US-IVD test in our study.

MTB detection with Ultra is defined as one or both of the probes that detect the multi-copy insertion elements *IS6110* and *IS1081* being positive, with Ct less than 37 and at least two *rpoB* probes with Ct less than 40 [20]. Ct values corresponding to the CFU/mL were plotted for *IS* (Figure 2E) and *rpoB* with four probes (Figure 2F–I) to visualize the relationship between Ct values and CFU/mL, with both the US-IVD and Ultra tests for each probe. With Ultra, the *IS* targets (Figure 2E) were more sensitive than the *rpoB* gene probes (Figure 2F–I). These regressions could be used to help predict the concentration (CFU/mL) of MTB in a sample based on the Ct values.

### 3.3. Semi-Quantitative Results by Ultra

**Figure 3:**
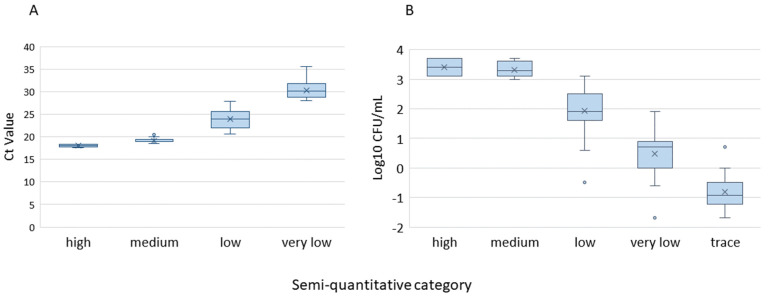
Semi-quantitative category reported on the Ultra test plotted against the lowest Ct values among the 4 *rpoB* gene probes for each sample (**A**) and the corresponding CFU/mL (log_10_) (**B**). The box represents the interquartile ranges from the lower quartiles (Q1) through the upper quartiles (Q3). The median and the mean are displayed as a solid horizontal line and × inside the box, respectively. The outliers are shown as data points outside the standard deviation bar.

The Ultra test is designed to report semi-quantitative results (high, medium, low, very low, and trace) corresponding to a Ct value range in one or more targets detected. For example, if one or both probes for the *IS* targets are positive with Ct less than 37 and no more than one *rpoB* probe has a Ct less than 40, Ultra calls it “MTB detected, trace” [20]. Here, we plotted the lowest Ct value among the four probes (B1, B2, B3, and B4) used for the *rpoB* gene for each sample versus the semi-quantitative categorical results (Figure 3A). All the samples tested in this study that had “trace” semi-quantitative results only had Ct values for the *IS* target, but not for any of the probes on the *rpoB* target. Therefore, the trace category was not included in Figure 3A. Figure 3B shows the CFU/mL in log scale versus the semi-quantitative categorical results. Figure 3 demonstrates the relationships between the amount of MTB in the specimens and the semi-quantitative categories.

## 4. Discussion

Overall, the analytical sensitivities of the Ultra test were substantially better than US-IVD for all sample types tested. Among the three sample types, BAL had the poorest analytical sensitivity on both the US-IVD and Ultra tests. BAL is not as homogenous as CSF and is more difficult to dilute than CSF, which may have accounted for some difference in the reported sensitivity. Since BAL and bronchoalveolar brush samples are typically considered superior non-sputum specimen types for MTB detection [6], we believe this is due to the higher organism burden in these specimen types clinically and not due to the sensitivity of the test. In contrast, pleural fluid is notoriously paucibacillary and therefore was not used to establish the analytical sensitivities of either test. In addition, Carnevale et al. reported that high cellular components and red blood cells could interfere with MTB detection in BAL and pleural fluid when certain extraction and amplification products were utilized [21]. While the pooled BAL matrix may have had moderate levels of interfering cellular contents, the breakdown of cellular components during storage may also have impacted the test performance and analytical sensitivity. Nonetheless, studies have shown that both Ultra and US-IVD can be used with BAL as an alternative to sputum for accurate detection of MTB in patients with low-yield sputum, smear-negative sputum, or tracheobronchial MTB [5,6]. Our results further demonstrated that both US-IVD and Ultra had even lower LoDs with BAL than sputum specimens in the experimental setting [9,12]. However, since these data were based on MTB prevalent regions, studies on the performance of Ultra on BAL samples compared to US-IVD in low prevalence regions are needed.

Lymphatic TB has consistently been the leading cause of EP TB in the US, based on the CDC data from 2010 to 2020 (https://www.cdc.gov/tb/statistics/surv/surv2020/default.htm, accessed on 7 August 2022). In our study, Ultra demonstrated the lowest LoD for MTB in lymph node tissues among all three sample types tested. Since we pooled lymph nodes from various anatomic locations, our data represent test performance on tissues from any lymphatic origin. However, since the pooled lymph node tissue was spiked post-homogenization, it does not truly represent patient lymph node samples containing organisms within the tissue. Therefore, the LoD for MTB in lymph node tissues reported in this study could be overestimated.

In the World Health Organization (WHO)’s report, US-IVD and Ultra are strongly recommended for establishing the diagnosis of meningeal TB from CSF samples [22]. There were discussions about the performance of US-IVD vs. Ultra for TB meningitis and whether one was more sensitive than the other. This could be explained by co-infection status with HIV and MTB prevalence in the population. A higher diagnostic accuracy from CSF samples was observed with Ultra in the HIV-negative group, whereas US-IVD performed better in HIV-positive group [23]. These differences were also likely influenced by whether or not the CSF was concentrated prior to testing [24].

In line with previous studies, the LoDs of MTB and RIF were lower (i.e., more sensitive) with Ultra than US-IVD in our study. This could also indicate that HIV-negative individuals with a lower bacterial burden than their counterparts would be more likely diagnosed by Ultra than US-IVD [17,24]. While WHO recommended using US-IVD for the initial diagnosis of TB meningitis, caution must be exercised in interpreting results depending on the disease prevalence in the region and underlying comorbidities, such as HIV status. Most studies describing the high accuracy of Ultra were performed in TB-prevalent areas. Although meningeal TB prevalence in the US was only 4% of the total EP TB per CDC’s report in 2020 (https://www.cdc.gov/tb/statistics/surv/surv2020/default.htm, accessed on 7 August 2022), the actual number of cases might be slightly higher due to possible misdiagnoses. Follow-up studies in the US entailing clinical correlations of TB meningitis and the use of Ultra in establishing the diagnosis are needed.

Because Ultra can detect MTB at “trace” levels, this feature can be helpful in establishing TB diagnoses in paucibacillary conditions. Studies have shown that the “trace” calls result in the early diagnosis of TB in smear-negative, culture-positive paucibacillary situations, leading to prompt treatments [25,26]. This highlights the significant clinical impact of Ultra’s “trace” feature in critical situations where the diagnosis could have been missed or delayed otherwise. The WHO report in 2019 also indicated that the TB diagnosis made from “trace” calls by Ultra should be based on defined criteria, such as EP TB, pediatric population, or underlying HIV infection, where the bacterial burden can be typically low [4].

Although only one MTB strain was used in this study, our findings confirmed that the analytical sensitivities of both US-IVD and Ultra were higher on non-sputum than sputum specimens [9,12]. In addition, Ultra had even lower LoDs than US-IVD for all sample types. These results suggest that using Ultra could result in more TB case detection rates in subjects with paucibacillary TB and EP TB, positively impacting WHO goals to eradicate TB.

## Figures and Tables

**Table 1:** LoD_95_ in CFU/mL for US-IVD and Ultra for each sample type.

Sample Type		US-IVD (95% CI)	Ultra (95% CI)
CSF	MTB	3.3 (2.2–11)	0.16 (0.095–0.52)
	RIF	4.6 (2.9–14)	2.7 (1.4–10)
BAL	MTB	45 (22–260)	0.65 (0.39–2.3)
	RIF	60 (28– 353)	7.5 (3.6–46)
Tissue	MTB	15 (8.0–49)	0.11 (0.077–0.29)
	RIF	23 (12–70)	12 (3.7–151)

## Data Availability

All raw data are available upon request.
